# Overcoming the phantoms of the past: Influence of predatory stimuli on the antipredator behavior of island pitvipers

**DOI:** 10.1371/journal.pone.0288826

**Published:** 2023-10-24

**Authors:** João Miguel Alves-Nunes, Adriano Fellone, Ivan Sazima, Otávio Augusto Vuolo Marques

**Affiliations:** 1 Instituto de Biociências, Programa de Pós-Graduação em Biodiversidade, Letras e Ciências Exatas, Universidade Estadual Paulista “Júlio de Mesquita Filho”, São José do Rio Preto, São Paulo, Brazil; 2 Laboratório de Ecologia e Evolução, Instituto Butantan, São Paulo, São Paulo, Brazil; 3 Instituto de Biologia, Museu de Biodiversidade Biológica, Universidade Estadual de Campinas, Campinas, São Paulo, Brazil; University of Regina, CANADA

## Abstract

The reduction of predation is a potentially important factor for the evolution of the traits of an island animal species. By relaxed selection, insular animals tend to lose their antipredator behaviors. A monophyletic group of pitvipers (genus *Bothrops*) in southeastern Brazil, which have high genetic affinity and dwell on the mainland and adjacent islands, provide an appropriate setting to study the evolution of antipredator behavior and how different predatory stimuli can influence this behavior. The mainland *Bothrops jararaca* has several terrestrial and aerial predators, whereas *B*. *insularis* and *B*. *alcatraz*, restricted to two small islands, Queimada Grande and Alcatrazes, respectively, have a smaller range of aerial predators. Terrestrial predators are absent on Queimada Grande, but one potential snake predator occurs on Alcatrazes. We observed that the defensive repertoire of island snakes has not been lost, but they display different frequencies of some antipredator behaviors. The type of predatory stimuli (terrestrial and aerial) influenced the defensive response. *Bothrops insularis* most often used the escape strategies, especially against terrestrial predatory stimuli. *Bothrops alcatraz* displayed the highest rate of strike for both terrestrial and aerial stimuli. Our results indicate that even though relaxed selection may occur in island environments as compared to mainland environments, these pitvipers still retain their antipredator behaviors but with different response degrees to the two predator types.

## Introduction

One of the most relevant interactions between animals in a community is the prey-predator relationship. Predation drives the development of several adaptations in animals. Among these adaptations, the behavioral ones deserve attention, especially the actions that avoid or lessen predation, i.e., the anti-predator behaviors [1]. Like many other animals, snakes have a wide variety of predators, both invertebrates and vertebrates, with different sizes, specialization degrees, capture tactics, metabolic rates, and life habits [2–4]. No single antipredator behavior fits all predation possibilities [5], even if snakes display an extensive defensive repertoire, one of the most elaborate described for reptiles [2].

Antipredator behavior is influenced by intrinsic factors such as age, sex, reproductive condition, and experience or learning as well as extrinsic factors as habitat, temperature, and social context [6,7]. Among these factors, the type of predator and the predation pressure it exerts has great influence [7]. Snakes respond in different ways depending on the type of predatory stimulus, which includes the predator’s color, size, and temperature [8–12]. When isolated from predators, expensive and no longer functional antipredator behaviors tend to be eliminated by relaxed selection [13–15]. Islands generally harbor few predators, as these environments usually have depleted fauna and do not house predators to the same extent as the mainland environments [16]. Therefore, it would be expected that some specific antipredator behaviors would be eliminated by selection if there were no benefits.

**Fig 1:**
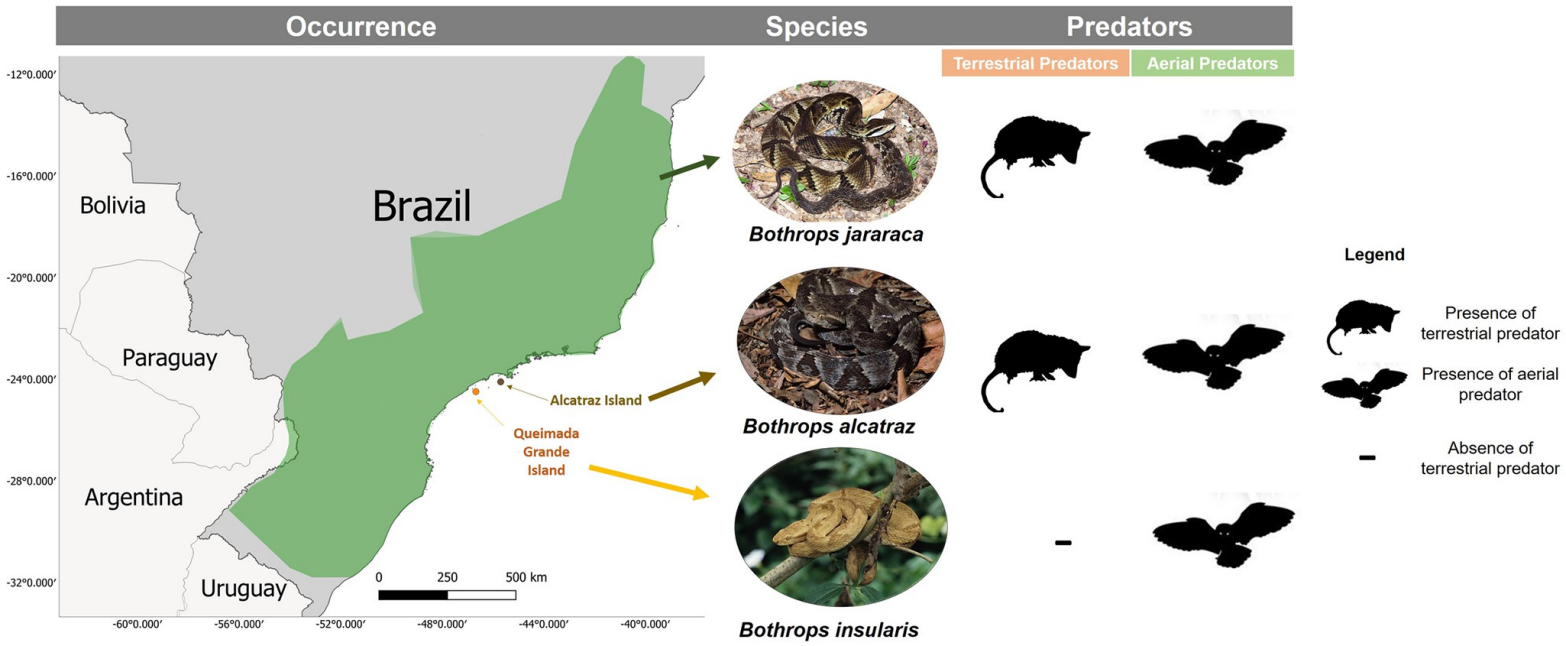
Predator types in the habitats of the three related pitviper species.

The Alcatrazes lancehead *Bothrops alcatraz* and the Golden lancehead *Bothrops insularis* are pitvipers endemic to Alcatrazes Island and Queimada Grande Island, respectively located about 30 to 35 km off the coast of the state of São Paulo, southeast Brazil [17–19]. Both species have similar origins [17–19]. During the Pleistocene, the sea level oscillated many times and the isolation of these islands from the mainland occurred about 11,000 years ago, splitting pitviper populations [17–19]. Genetic studies show that both *B*. *alcatraz* and *B*. *insularis* bear great similarity to the mainland pitviper *Bothrops jararaca* [17–20]. The three species experience different predatory pressures: *Bothrops jararaca* occurs in a wide range of forested habitats on the continent and consequently faces a wider range of predators, both aerial and terrestrial [21,22], whereas *B*. *alcatraz* and *B*. *insularis* are restricted to small area islands [17–19] with different predation profiles (Fig 1). The Queimada Grande Island has an area of 43 ha with no terrestrial predators, but houses 56 forest bird species, some of which are potential snake predators [18,22]. The Alcatrazes Island has an area of 135 ha and houses a potential terrestrial predator (the large lizard *Salvator merianae*) and 76 bird species, some of which are potential snake predators [17,18,22,23].

These three pitviper species with their respective evolutionary and ecological histories provide an appropriate setting to study the evolution of antipredator behavior, and how different predatory stimuli can influence this behavior. Our aim is to identify the influence that predatory stimulus types have on antipredator behavior in island pitvipers compared to the mainland. Specifically, to evaluate the influence of terrestrial and aerial predatory stimuli on the frequency of each behavior in the antipredator repertoire of each of the three pitviper species. Due to the different set of predators on the two islands and on the mainland, our working hypothesis is that some behavioral reactions of island snakes will be absent or distinct from those displayed by the mainland snake. Additionally, we predict that, due to the different types of predators on each island, the two island pitvipers differ in their defensive behaviors.

## Materials and methods

### Ethics

All procedures were approved by the Ethics Committee on Animal Use of the Butantan Institute (CEUAIB) and are in accordance with the guidelines of the National Council for the Control of Animal Experimentation (CONCEA) for the care and use of animals in research (CEUA 2705070821).

### Study animal

We used 47 snakes, of which 20 *B*. *jararaca* individuals (10 males and 10 females), 19 *B*. *insularis* individuals (10 males and 9 females) and 8 *B*. *alcatraz* individuals (3 males and 5 females). All snakes were born in captivity, from different mothers, and were housed in the Laboratory of Ecology and Evolution at the Instituto Butantan. This procedure removes the effect of previous experience that each individual could have experienced in natural habitats [24]. Although the behaviors of captive animals can be influenced by habituation to laboratory conditions, Araújo & Martins [25] demonstrated no differences between some anti-predator behaviors of captive and wild *Bothrops* species. All snakes used were adult (i.e., sexually mature) using snout-vent length (SVL) data [26,27]. The snakes were kept at a temperature around 25°C, under a light and dark photoperiod of 12: 12 h.

### Behavioral tests

To perform the behavioral tests, we conducted confrontations with the snakes using aerial and terrestrial predator models as stimuli (Supplementary material). The tests were conducted at night (between 6 PM and 10 PM), under artificial lighting. For the confrontations, a 2.025 m^2^ [1.5m (length) X 1.35m (width) X 1m (height)] arena was set up, with an aluminum plate as a base, and Styrofoam walls. All experiments were carried out between 5 pm and 8 pm at a temperature around 23°C. For each behavioral test, the snakes were left in the arena for up to 15 minutes in order to cease exploratory behaviors and habituate to the test arena.

To simulate a terrestrial predator, we used a cloth and cotton model of the white-eared opossum (*Didelphis albiventris*) as this mammal preys on *B*. *jararaca* [28]. A bottle with water at 37°C was placed inside the model to simulate the typical internal temperature of mammals. Using a 1.5 m wooden stick attached to the predator model, 30 advances of the model toward the snake’s head were performed with a 2 s interval, touching the snake’s body. To simulate an aerial predator, we used a taxidermied burrowing owl (*Athene cunicularia*) with spread wings. This owl species preys on pitvipers, besides occurring on the two islands [22]. At each behavioral test, the model was warmed with the help of heaters so its temperature was around 37°C to 39°C. With the help of a 2 m long fishing rod, the owl model was attached and confronted the snake 30 times with a 3 s interval, touching the snake’s body. These confrontations were performed from the top downwards simulating an aerial attack. We touched the snake from a distance of about 3 m from the observer at each test and an interval of seven days between the tests to avoid learning and habituation influences [24]. Both predator models had their irradiation temperature measured using an infrared thermometer (ST-620, Incoterm) before and after each behavioral test, ensuring that the models had similar temperatures to mammals and birds. All tests were recorded with a film camera (HDR-PJ200, Sony) for later analysis.

**Fig 2:**
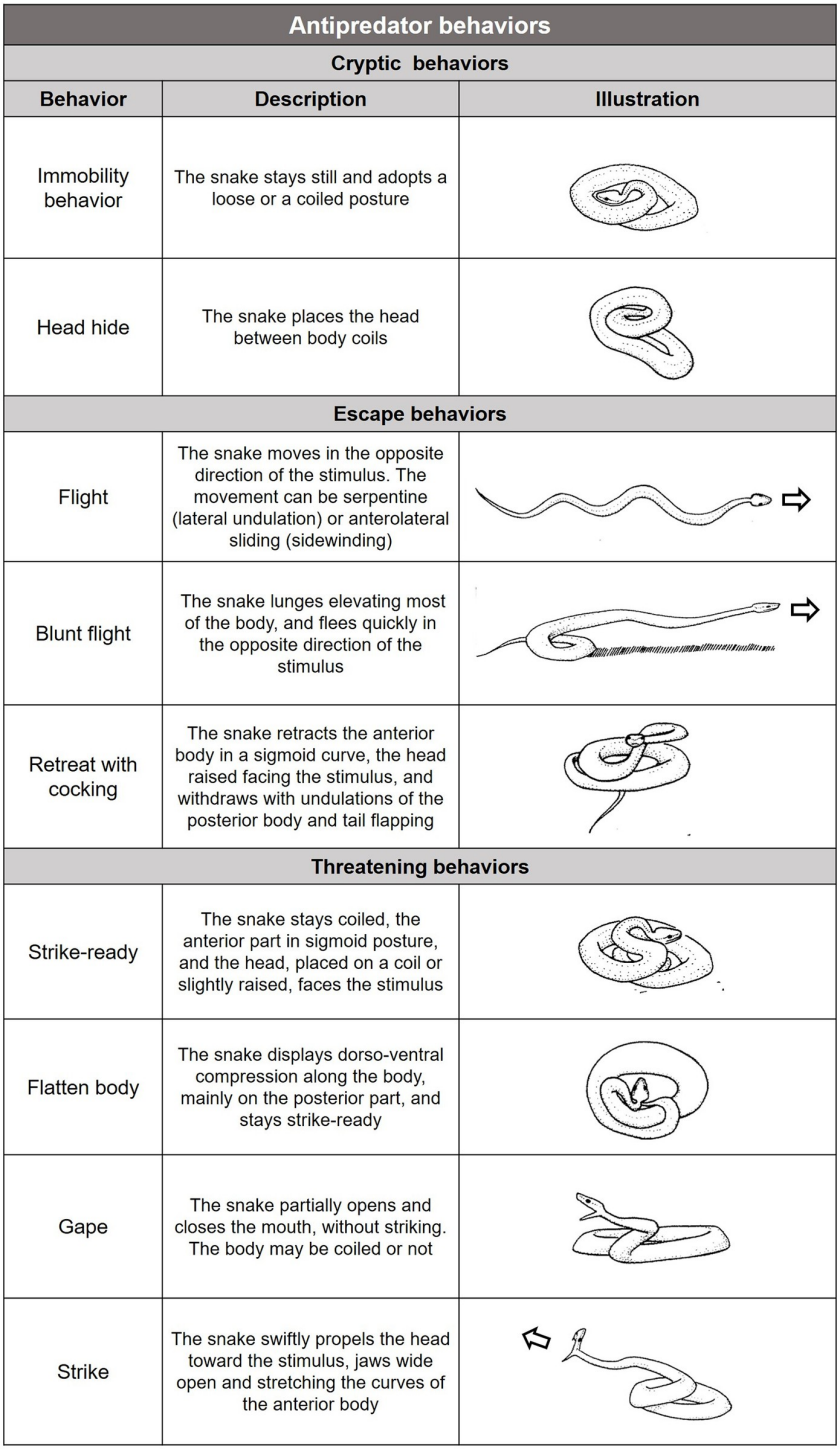
Antipredator behaviors of the pitvipers and their explanation. Descriptions and illustrations partly based on Greene [2], Sazima [29] and Araújo & Martins [25].

The behaviors displayed by the snakes during the tests were classified by two researchers independently and analyzed for congruence according to Greene [2], Sazima [29], and Araújo & Martins [25] (Fig 2). In addition, following Mori and Burghardt’s [8] apparent function behavioral classification, we grouped the behaviors into three classes to simplify understanding the results. Cryptic behaviors (immobility and head hiding) are an attempt to remain unnoticeable to the predator. Escape behaviors (flee; blunt escape, and cocking) increase the distance between the snake and the predator. Threatening behaviors (gaping; body flatten; strike-ready and strike) are responses that include any behavioral element to prevent or lessen predation by displaying a potential danger to the predator.

### Statistical analysis

The fixed categorical predictor variables were snake species (three levels: *B*. *alcatraz*, *B*. *insularis*, and *B*. *jararaca*) and predatory stimuli (two levels: terrestrial and aerial predator models). The response variables were the frequency of each antipredator behavior during the 30 confrontations with each predator model. Due to the nature of these variables and based on the experimental design, we used a generalized linear mixed model (GLMM) with Poisson distribution and log link function for each behavior. Only for body flattening a GlmmTMB model with a negative binomial distribution (nbinom1) was used. The model consisted of the additive and interactive property between the variables (species and predator). As we conducted the tests with the same individual snakes, and the sex was not of interest for our investigation, we included these two variables (ID and sex) as random variables in the models, thereby eliminating pseudoreplication in the experimental design. In addition, we performed Tukey’s test for post-hoc analysis to find significant relationships between the variables, using the "emmeans" package. All models were subjected to data dispersion analysis, homoscedasticity, and delineate tests using model diagnostic values and plots, with the help of the package "DHARMa: residual diagnostics for hierarchical (multilevel/mixed) regression models" in R (version 4.04).

## Results

### Cryptic behaviors

**Fig 3:**
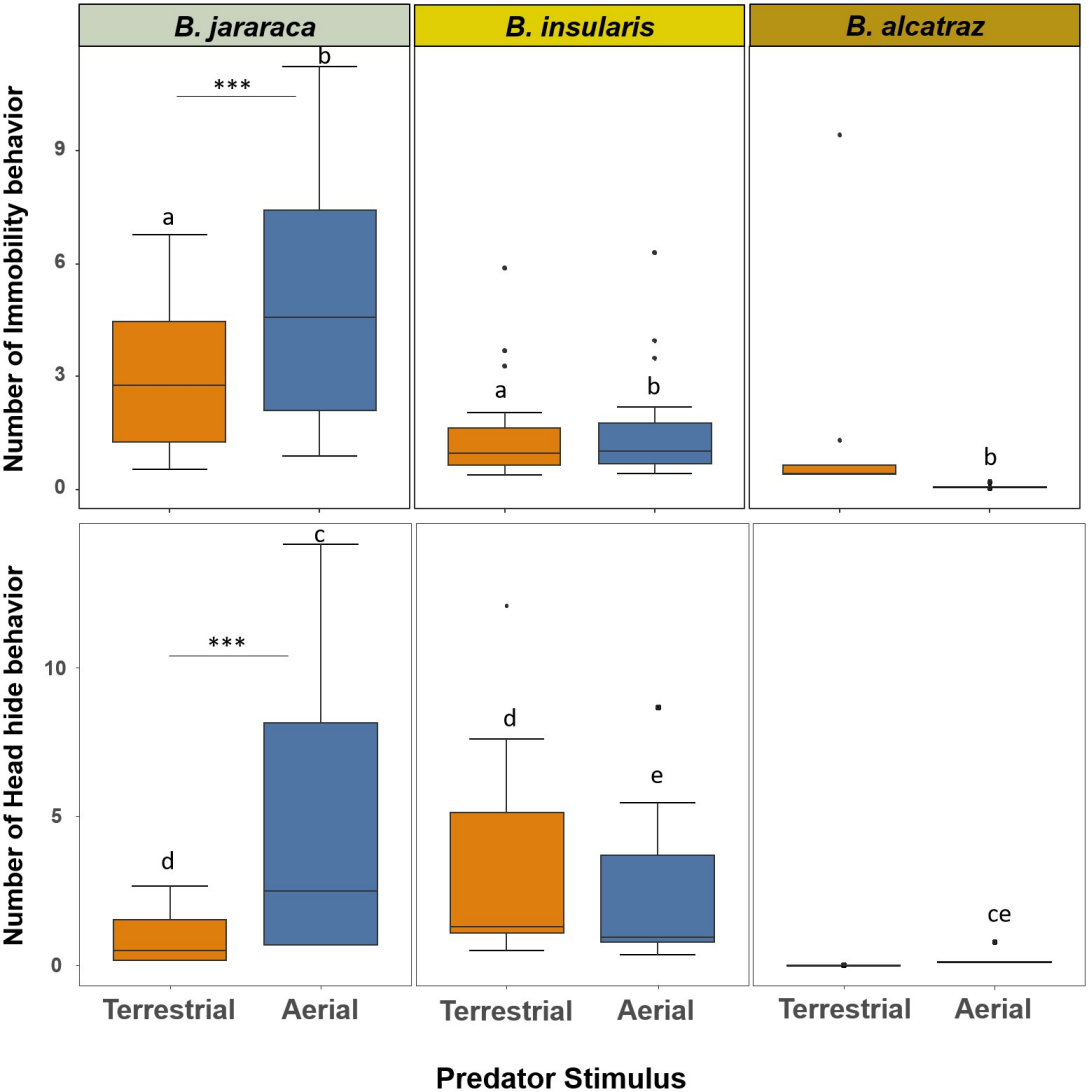
Influence of predatory stimulus types on cryptic category behaviors of *Bothrops jararaca*, *B*. *insularis* and *B*. *alcatraz*. Boxplot graph representing the dispersion and asymmetry of groups of data. *Box*: Represents the second and third quartiles and a central line (median). *Whiskers*: The lines extending from the box indicate the dispersion of the data, excluding discrepant values. *Outliers*: The points beyond the whisker’s boxes represent outliers or extreme values in the data distribution. *** indicates that the results show a clear difference in the frequency of behaviors in relation to the type of predatory stimulus among each species (p < 0.001). Corresponding lowercase letters indicate a significant difference between groups (p < 0.05).

The three pitivipers differed in the frequency of cryptic behaviors such as immobility and head hiding, *Bothrops alcatraz* showing the least tendency to display cryptic behaviors (Fig 3). Predatory stimulus influenced immobility behavior for *B*. *jararaca* only, this species showing a greater tendency to immobilize to aerial predator than to terrestrial predator stimulus by 40% (z = 3.234, p = 0.00122) (S1 Table). In addition, *B*. *jararaca* showed the highest rate of immobility among the studied species (Fig 3, S2 Table). The only pitviper species that showed a clear difference in the frequency of head-hiding behavior when exposed to different stimuli was *B*. *jararaca* (*B*. *jararaca*: z = 6. 349, p < 0.001; *B*. *insularis*: z = 1.667, p = 0.5535; *B*. *alcatraz*: z = -0.147, p = 1.000) (S3 and S4 Tables). In addition, *B*. *jararaca* hid its head more often from aerial predator stimulus than *B*. *alcatraz* (z = 3.053, p = 0.0275). However, *B*. *insularis* exposed to terrestrial predatory stimulus had a higher rate of head-hiding behavior than *B*. *jararaca* (z = 2.792, p = 0.00524) (S4 Table).

### Escape behaviors

**Fig 4:**
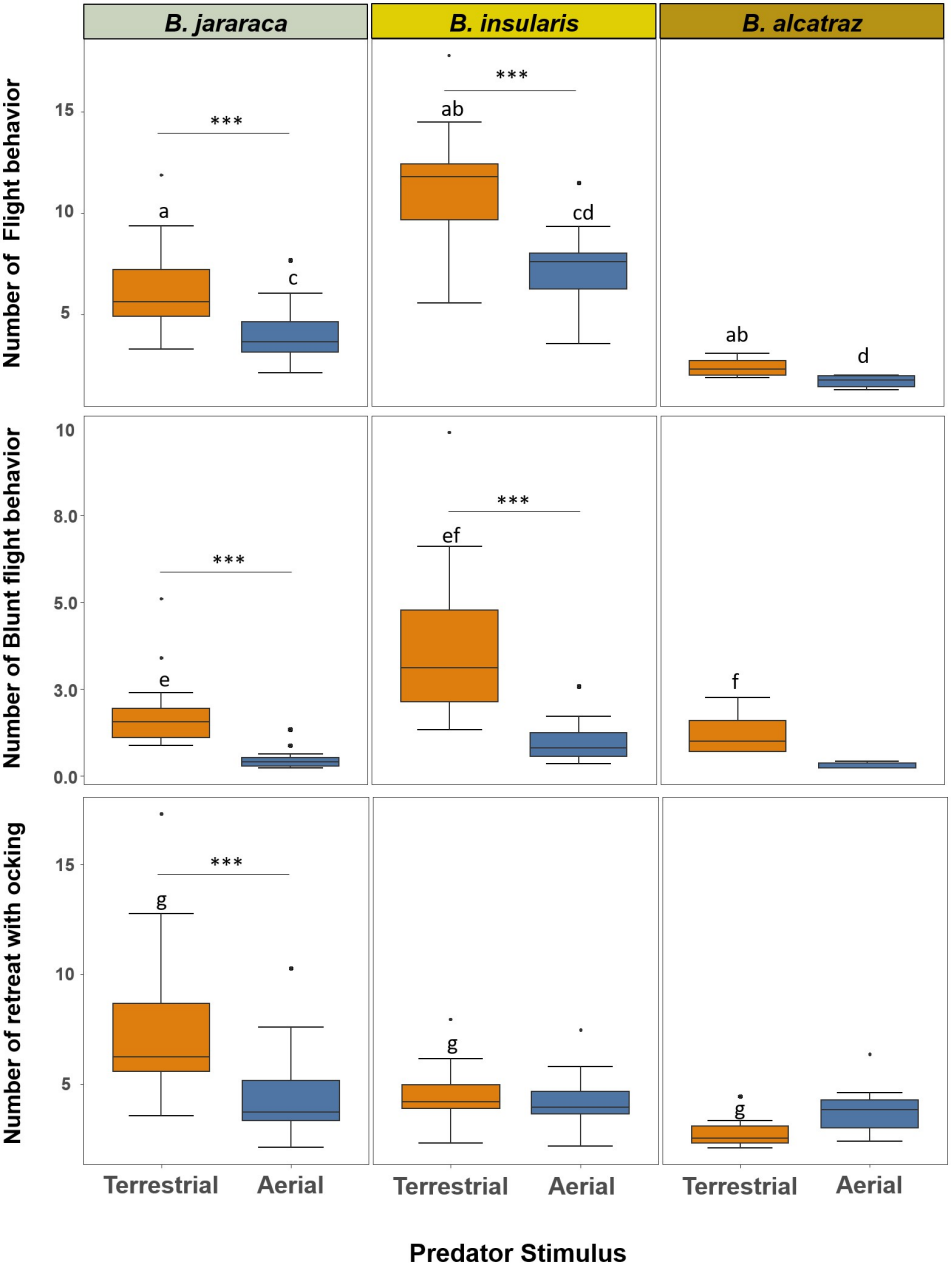
Influence of predatory stimulus types (terrestrial and aerial) on escape category behaviors of *Bothrops jararaca*, *B*. *insularis* and *B*. *alcatraz*. Boxplot graph representing the dispersion and asymmetry of groups of data. *Box*: Represents the second and third quartiles and a central line (median). *Whiskers*: The lines extending from the box indicate the dispersion of the data, excluding discrepant values. *Outliers*: The points beyond the whisker’s boxes represent outliers or extreme values in the data distribution. *** indicates that the results show a clear difference in the frequency of behaviors in relation to the type of predatory stimulus among each species (p < 0.001). Corresponding lowercase letters indicate a significant difference between groups (p < 0.05).

Predatory stimulus type strongly influenced the escape behaviors. Both *B*. *jararaca* and *B*. *insularis* fled more frequently when confronted with a terrestrial predator stimulus (opossum model) than with an aerial predator (owl model) (*B*. *jararaca*: z = -3.458, p = 0.0005, *B*. *insularis*: z = 4.288, p = 0.0003) (Fig 4, S5 and S6 Tables). *Bothrops insularis*, independently of the predatory stimulus, showed a higher flight rate than the other species. For the stimulus of an aerial predator, flight probabilities were found to be approximately 47% and 68% higher than those of *B*. *jararaca* and *B*. *alcatraz*, respectively (*B*. *insularis—B*. *jararaca*: z = -3.474, p = 0.0068; *B*. *insularis—B*. *alcatraz*: z = 3.972, p = 0.0010) (S6 Table). In the case of terrestrial predator stimulus, chances of flight were observed to be 46% and 85% greater compared to *B*. *jararaca* and *B*. *alcatraz*, respectively (*B*. *insularis—B*. *jararaca*: z = -3.786, p = 0.0021; *B*. *insularis- B*. *alcatraz*: z = 6.062, p < 0.0001) (S6 Table).

Similarly to the previous behavioral category, only *B*. *alcatraz* did not have its blunt flight frequency influenced by the predator type (*B*. *jararaca*: z = -3.295, p = 0.0010; *B*. *insularis*: z = -5.499, p < 0.0001; *B*. *alcatraz*: z = 1.635, p = 0.5753 (Fig 4) (S8 Table). *Bothrops jararaca*, regardless of the predatory stimuli, had no clear difference in frequency when compared to *B*. *alcatraz* (terrestrial: z = 0.872, p = 0.9532; aerial: z = 0.675, p = 0.9847). However, *B*. *insularis*, once again, displayed higher frequencies of blunt flight compared to the other species, with approximately 54% and 70% higher likelihood than *B*. *jararaca* and *B*. *alcatraz*, respectively (*B*. *insularis- B*. *jararaca*: z = -2.470, p = 0.0135; *B*. *insularis- B*. *alcatraz*: z = -2.579, p = 0.0099) (S8 Table).

Cocking behavior showed different patterns depending on the pitviper species and predatory stimuli (Fig 4), and the dependence between variables was significant. For instance, *B*. *insularis* and *B*. *alcatraz* showed no difference towards the type of predatory stimulus (*B*. *insularis*- z = 0.391, p = 0.9988; *B*. *alcatraz*- z = -1.169, p = 0.8516), but different predatory stimuli influenced the frequency of cocking by *B*. *jararaca* (z = -3,936; p < 0.0001) (S9 Table). In addition, when exposed to the terrestrial predator stimulus *B*. *jararaca* showed the highest frequency of retreat with cocking among the three pitvipers (*B*. *jararaca-B*. *insularis*: z = -2.408, p = 0.01604; *B*. *jararaca-B*. *alcatraz*: z = -3.515, p = 0.00044) (S9 and S10 Tables).

### Threatening behaviors

All behaviors involving a threat signal, such as body flatten, gape, strike-ready, and strike were influenced by the type of predatory stimulus among the three pitviper species.

**Fig 5:**
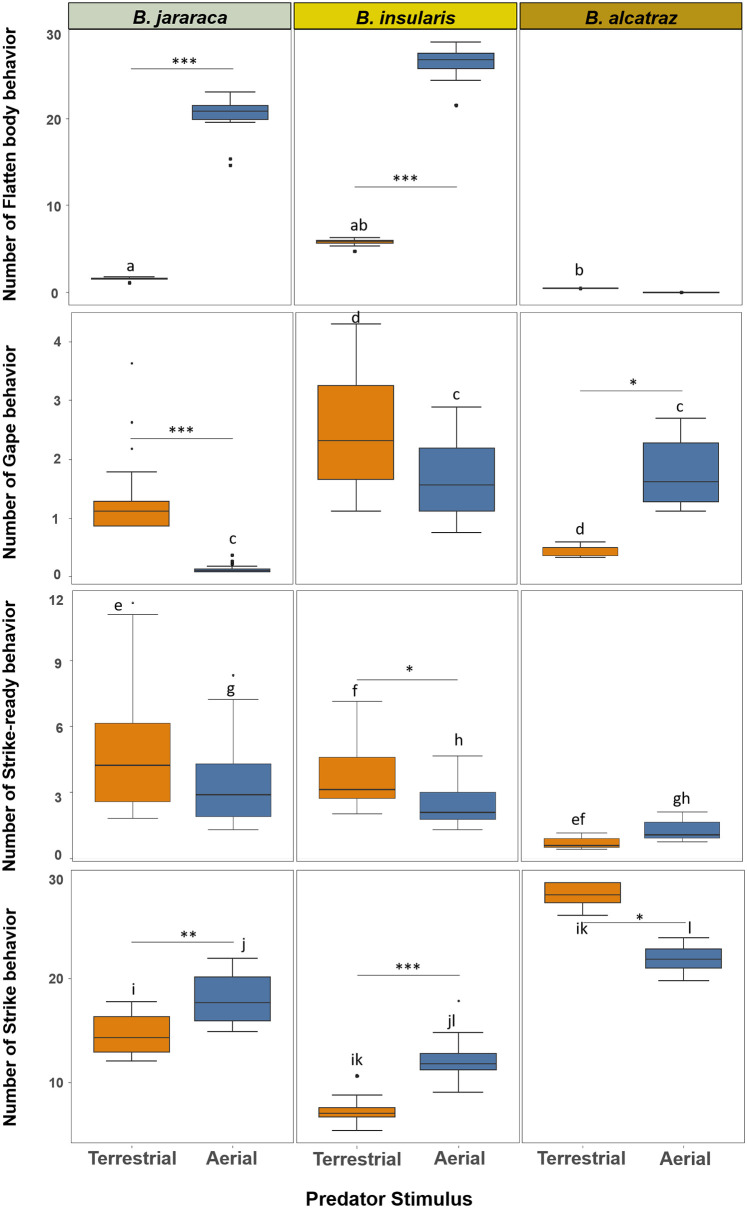
Influence of predatory stimulus types on escape threatening behaviors of *Bothrops jararaca*, *B*. *insularis* and *B*. *alcatraz*. Boxplot graph representing the dispersion and asymmetry of groups of data. *Box*: Represents the second and third quartiles and a central line (median). *Whiskers*: The lines extending from the box indicate the dispersion of the data, excluding discrepant values. *Outliers*: The points beyond the whisker’s boxes represent outliers or extreme values in the data distribution. Asterisk: Indicates that the results show a clear difference in the frequency of behaviors in relation to the type of predatory stimulus among each species (*** p < 0.001; ** p < 0.01; * p < 0.05) Corresponding lowercase letters indicate a significant difference between groups (p < 0.05).

Flatten body was strongly influenced by the type of predator for two of the studied pitvipers. *Bothrops alcatraz* had a different behavioral pattern than the other species and did not show body flattening. On the other hand, both *B*. *jararac*a and *B*. *insularis* displayed more body flattening when faced with the aerial predatory stimulus than the terrestrial one (z = -6.638, p < 0.0001). Moreover, *B*. *insularis* flattened its body the most among the three species, mainly to terrestrial predator stimulus (*B*. *jararaca-B*. *insularis*: z = 3.169, p = 0.00153; *B*. *insularis- B*. *alcatraz*: z = -2.527, p = 0.0115) (Fig 5; S13 and S14 Tables).

The predator stimulus type strongly influenced gape behavior in all three species, more pronounced in *B*. *insularis*. *Bothrops jararaca* and *B*. *insularis* gaped more frequently towards the terrestrial predator stimulus (z = -3.803; p = 0.000143), although the difference was not significant for the latter pitviper (z = -1.905; p = 0.056742). On the other hand, in *B*. *alcatraz* gape was elicited more often by the aerial predatory stimulus (z = 2.265; p = 0.02352) (Fig 5). Furthermore, *B*. *jararaca* showed less frequency of gape behavior with aerial predator stimulus than the other species (*B*. *jararaca- B*. *insularis*: z = -3.920, p = 0.0012; *B*. *jararaca- B*. *alcatraz*: z = -3.814; p = 0.0019) (S15 and S16 Tables). *Bothrops insularis* exposed to the terrestrial predator stimulus showed the highest frequency of this behavior among the three species (z = 2.896, p = 0.0439).

Strike-ready behavior had a similar pattern for *B*. *jararaca* and *B*. *insularis*, with a more pronounced reaction to the terrestrial predator stimulus than the aerial one. Although only *B*. *insularis* showed a significant difference (z = -2.257, p = 0.02398), the p-value for the difference in the reaction of *B*. *jararaca* was marginally significant (z = -1.853, p = 0.0639). No difference in this behavior was detected *in B*. *alcatraz* (z = -0.988, p = 0.3231). However, this pitviper displayed the least strike-ready reaction towards the terrestrial predator stimulus among the three species (*B*. *alcatraz*: *B*. *jararaca*: z = 3.710, p = 0.000207; *B*. *alcatraz*: *B*. *insularis*: z = 3.316, p = 0.000915) (S11 and S12 Tables).

Strike was another behavior strongly influenced by the predatory stimulus type. *Bothrops jararaca* and *B*. *insularis* showed the same pattern, with more strikes towards terrestrial than aerial predators stimuli (*B*. *jararaca*: z = 2.684, p = 0.007273; *B*. *insularis*: z = 4.860, p < 0.0001) (S17 Table). In contrast, *B*. *alcatraz* showed the opposite behavior. When faced with the predator stimuli, it displayed more strikes towards the aerial than the terrestrial stimulus (z = -2.217, p = 0.026609). Among the three pitviper species, *B*. *alcatraz* displayed the highest strike behavior rates (Fig 5). The odds of *B*. *alcatraz* to strike a terrestrial predator were about 75% and 49% higher than *B*. *insularis* and *B*. *jararaca*, respectively. While for an aerial predator, *B*. *alcatraz* had about 44% more odds than *B*. *insularis* to display strike behavior (z = -4.715, p < 0.0001). When compared to *B*. *jararaca* the difference of 27% chance is not significant (z = -1.604; p = 0.108618).

## Discussion

Our study simulating aerial and terrestrial predators demonstrated that these two contrasting predatory stimuli influenced the frequency of each category of antipredator behavior displayed by the three pitviper species. Furthermore, we showed that the mainland *B*. *jararaca* and its closest island species reacted diversely to the same predatory stimulus. Our results do not support the hypothesis that the antipredator behavior repertoire of island species was lost due to isolation. However, a quantitative difference in the defensive behaviors displayed by each snake species was evident. *Bothrops insularis* was the species that displayed the highest frequency of escape behaviors, mainly towards terrestrial predators. *Bothrops jararaca* displayed more cryptic behaviors among the three pitviper species, the more so towards aerial stimuli. On the other hand, *B*. *alcatraz* was the species most prone to bite towards both stimuli types.

A previous study on defensive behavior of five *Bothrops* species found quantitative differences in behavioral categories that seem to be adaptive reactions to variable predation pressure of aerial predators, as each of the five studied pitvipers dwells in a distinct habitat [29]. However, it must be emphasized that snakes can change their antipredator behavior during a snake’s life depending on previously experienced predation pressure [30]. Adult snakes exposed to a higher risk of predation showed higher bite rates, whereas there was no such association among newborns [30]. All snakes used in our study were born in captivity, had no previous contact with natural predators, and would not change behaviors by ontogeny or plasticity.

Among the escape behaviors we recorded for the three pitvipers, cocking was the most variable. Escape behaviors are strategies that increase the distance between a snake and a potential predator [8], and generally, the snake directs its head to the opposite side of the predatory stimulus. However, cocking is the only behavior that, while increasing the distance between the snake and the stimulus object, the snake keeps its head facing the potential predator. *Bothrops jararaca* was the only species that displayed changes in frequency of cocking depending on the predatory stimulus. Possibly, this influence is due to the greatest diversity of predators sympatric with *B*. *jararaca*, and cocking behavior may be important to avoid predation by a predator type that the other pitvipers do not come across [31]. For the other escape behaviors (flight and blunt flight), all species displayed a similar pattern, which was more frequent when confronted with terrestrial predators. Possibly this is due to this predator type being on the same physical plane as the snake, and can attack more efficiently than an aerial predator. Therefore, snakes would tend to evade as quickly as possible, as behaviors that include moving away from the predatory stimulus are the main ones used by snakes [7,32].

The frequent flight and blunt escape are the defensive traits of *B*. *insulari*s that differentiates it most from the other two pitvipers. There is an evident predator low richness on Queimada Grande Island and experiments with plasticine snake replicas showed a significantly lower number of attacks on this island than in continental sites [18]. Our hypothesis is that this low predatory pressure on the island reduces the encounter rate with any potential aversive or non-aversive stimulus (terrestrial or aerial), which lowers the sensitivity threshold to respond to something disturbing. However, *B*. *jararaca*, which dwells in areas with higher species richness, would tend to be more habituated (with a higher sensitivity threshold for response) to possible stimuli, making it more adaptive to remain motionless and evaluate the predatory stimulus rather than fleeing and thus standing out from the substrate. Consequently, the frequency of other defensive behaviors in the defensive repertoire of *B*. *insularis* is reduced compared to those of mainland *B*. *jararaca*, making fleeing the main defense strategy of this pit viper on the island. Moreover, the low predator pressure could also explain the uniform yellowish coloration of this island species, apparently out of tune with the environment. The higher frequency of escape behavior displayed by *B*. *insularis* may also be explained by is its distinctive coloration. Striped or differently colored snakes have a tendency to escape more frequently than those with spotted coloration, which tend to be more cryptic and stationary as their coloration pattern favors camouflage [33]. Based on this premise, *B*. *alcatraz* and *B*. *jararaca* with their chevroned pattern should display cryptic behaviors more often than *B*. *insularis*, but we found that *B*. *alcatraz* did not display any cryptic behavior.

We found that all three pitviper species tend to use cryptic behaviors more towards aerial than towards terrestrial predator stimulus, although this was less evident for *B*. *alcatraz*. Absence of movements lessens the detection of a snake by aerial predators, as birds perceive their prey visually mostly by movements of the prey [34]. Furthermore, *B*. *jararaca* displayed head hiding more often towards aerial predators than terrestrial predators. Birds of prey, such as the snake-specialist Laughing falcon (*Herpetotheres cachinnans*), kill their snake prey pecking at its head or tearing the head off [35]. Snakes tend to hide their most vital part (head) during aerial attacks with higher head attack rates [31].

Threat behavior would warn the predator that its potential prey is dangerous [2,8]. Within threat behavior class, strike is the one most influenced by abiotic and biotic factors [8,11,36]. Both *B*. *jararaca* and *B*. *insularis* displayed higher strike frequency towards aerial stimuli than to terrestrial ones. This tendency of *B*. *insularis* is likely influenced by the lack of terrestrial predators on the island, whereas for *B*. *jararaca* it could be due to greater pressure from predatory birds than from predatory mammals (29). On the other hand, *B*. *alcatraz* displayed more threatening behaviors towards terrestrial predators, possibly due to this pitviper being under greater predation pressure by terrestrial predators than the aerial ones. The large tegu lizard (*Salvator merianae*) is abundant on the island [18,37], and is able to track and prey on rats and smaller lizards [38,39], which means that it would be a potential snake predator. Among the three species, *B*. *alcatraz* displayed the highest strike frequency. This species is very small and shares similarities with juvenile *B*. *jararaca*, as it has pedomorphic traits [17,40]. Snake size is one of the main variables that interfere with strike behavior [7,10]. Being more susceptible to predation pressure, small snakes tend to have higher strike rates [29,41–45], which would explain the marked defensive behavior of *B*. *alcatraz*. Another important factor is that we have no knowledge of the aerial predation frequency, although there are more potential avian predators on Alcatrazes island than on Queimada Grande island, even though far less than on the mainland [18]. A behavior closely linked to strike is strike-ready. Unexpectedly, *B*. *alcatraz* displayed almost no strike-ready behavior towards the predator stimuli. Possibly, higher predation risks on this small pitviper would lead to few strike-ready warnings and more strikes, a hypothesis that merit verification.

Another threatening behavior is gape. *Bothrops insularis* used this behavior most often among the three pitvipers. Gape behavior is used by arboreal and diurnal snake species [3] and the Golden Lancehead is the most arboreal species among the three studied pitvipers [17,46]. In addition, *B*. *insularis* is more diurnal than the two other species, which are predominantly nocturnal [47,48]. Both *B*. *insularis* and *B*. *jararaca* displayed more gape behavior towards terrestrial predator stimulus than towards the aerial one. Gape is a display that increases the size of the snake on an x-axis, and is possibly more effective for predatory confrontations that occur on the same spatial plane. Both *B*. *jararaca* and *B*. *insularis* showed very similar patterns of threat behaviors. However, *B*. *alcatraz* showed a pattern opposite to the other two species. Tests comparing *B*. *jararaca* juveniles and adults could indicate whether this difference is also due to pedomorphic traits. Field observations showed that *B*. *jararaca* juveniles and males are more prone to strike than adult female individuals (47). Furthermore, we emphasize that *Bothrops jararaca* and *B*. *insularis* come from the same *Bothrops* population lineage, whereas *B*. *alcatraz* comes from another *B*. *jararaca* population lineage [19].

Flatten body behavior expands the snake’s body laterally [2], and when viewed from above the snake appears larger. Bird attacks occur overhead and often during daylight [34]. Both *B*. *jararaca* and *B*. *insularis* flattened more when confronted with aerial predatory stimuli than with the terrestrial one. Unexpectedly, this behavior was not recorded for *B*. *alcatraz* for any predator type stimuli. Our small sample does not allow us to say that *B*. *alcatraz* has lost this behavior, but at least it seems to be less frequent in this species. *Bothrops insularis* hunts during the day and *B*. *jararaca* is also observed in daylight with some frequency [17,18,46]. On the other hand, *B*. *alcatraz* seems to be more secretive during the day, being usually found within a decomposing trunk or under a fallen leaf [18].

Antipredator behavior can be lost after the loss of a key predator, i.e., loss of a trait after relaxed selection [49], or it can persist for many generations [50]. If unnecessary antipredator behavior has substantial costs when displayed, then loss of predators should lead to rapid trait loss [51]. Thus, island pitviper species that usually have lower predation rates than mainland pitvipers would tend to lose or decrease the frequency of some antipredator behaviors. Essentially, we found that the defensive behavior repertoire persists in island pitvipers but their frequency changes. As emphasized above, the only exception was the absence of flattening in *B*. *alcatraz* (apparently lost). The persistence of antipredator behaviors in the three studied pitvipers is consistent with the multipredator hypothesis. This hypothesis attempts to explain the persistence of antipredator behavior under relaxed selection [52]. According to Blumstein [52], the hypothesis emphasizes that antipredator behavior has pleiotropic effects on other traits, which will remain functional regardless of the presence or absence of predators. This implies that a gene associated with defensive behavior can have multiple effects. For example, genes related to strike behavior would be preserved even in the absence of predators, as strike plays a fundamental role in other behaviors such as prey capture. Once this set of genetic behavioral characteristics is established, genes linked to defensive behavior are not lost, even in the absence of a specific predator. Thus, the diversity of predators or the existence of different functional demands would be factors favoring the maintenance of these genetic behavioral traits over time.

The multipredator hypothesis has been studied in some kangaroo and wallaby species (Diprotodontia), in which the loss of all predators apparently led to a rapid loss of antipredator behavior, while the loss of only one or two predators had a limited effect on the expression of antipredator behavior for the missing species [16,50,51]. The evolutionary persistence of the rattlesnakes (*Crotalus* spp.) recognition by California ground squirrels *Spermophilus beechey*i (Rodentia) [53,54] indicates that rattlesnake recognition ability can be maintained for over 70,000 years after rattlesnake isolation. *Bothrops insularis* and *B*. *alcatraz* were isolated from the mainland 11,000 years ago [17–19,55]. Therefore, the integrity of the antipredator behaviors of the isolated island species, based on the multi-predator hypothesis, should be due to some type of predator still exerting pressure on these populations or, if not, they are still relatively young population lineages and would not yet have lost the defensive repertoire.

Another explanation that may clarify the permanence of antipredator behavior is the "phantom predator past hypothesis" [50,56]. This hypothesis states that a species subjected to past selection for antipredator behavior by a predator that exerted strong predatory pressure will maintain antipredator behavior if it is not too costly [56,57]. The ancestral opossums (Didelphimorphia) have been in South America for at least 10 million years and the ancestral lineage of the genus *Didelphis* for 3 million years [58]. These mammals are important predators of snakes, including pitvipers, as they are resistant to toxins in the venoms [59,60]. Several birds, including raptors, also have been actively preying on snakes on the mainland for millions of years [61,62]. Therefore, even though some of these predators do not occur on the islands, they possibly exerted a strong predation pressure and selection of defensive behaviors on the ancestral lineages of the South American pitvipers since the early Pleistocene, which persists in the current lineages. Other behaviors, such as prey handling by *B*. *jararaca* and *B*. *insularis*, remain the same while preying on rats, despite the insular species being isolated for more than 11,000 years without access to this prey type [63]. Therefore, we can suppose that, like the feeding behavior conserved in these two species with phylogenetic proximity, the defensive behavior may have been preserved as well.

Our study is the first to investigate the antipredator behavior of island pitviper species in South America and is instructive for the fields of behavioral ecology and evolution of animal behavior. The two island species are endemic and critically endangered. Among the three species, *B*. *alcatraz is* the least studied, and this is the first study to investigate some ecological and behavioral aspects of this small species. Further comparative behavioral studies of these three species would be useful, as well as to study other island *Bothrops* species recently described [64,65]. Experimental studies with snakes in nature would be useful for a better understanding of antipredator behavior, providing information for the conservation of these island species. We encourage integrative studies on natural history (e.g., antipredator behaviors), evolution, and ecology that may help us understand better the behavioral strategies used by a snake species.

## Supporting information

S1 Fig Models of a terrestrial predator (Opossum: *Dideplphis albiventris*) and an aerial predator (taxidermied owl: *Athene cunicularia*). (DOCX)

S1 Table Summary of the model for immobility behavior. Intercept- Species (*B*. *jararaca*) and Predator (terrestrial). Bold p-values indicate p < 0.05.
(DOCX)

S2 Table Result of the comparison between groups for immobility behavior (predator and species) by Tukey’s test. Bold p-values indicate p < 0.05.
(DOCX)

S3 Table Summary of the model for head hide behavior. Intercept- Species (*B*. *jararaca*) and Predator (terrestrial). Bold p-values indicate p < 0.05.
(DOCX)

S4 Table Result of the comparison between groups for head hide behavior (predator and species) by Tukey’s test. Bold p-values indicate p < 0.05.
(DOCX)

S5 Table Summary of the model for flight behavior. Intercept- Species (*B*. *jararaca*) and Predator (terrestrial). Bold p-values indicate p < 0.05.
(DOCX)

S6 Table Result of the comparison between groups for flight behavior (predator and species) by Tukey’s test. Bold p-values indicate p < 0.05.
(DOCX)

S7 Table Summary of the model for blunt flight behavior. Intercept- Species (*B*. *jararaca*) and Predator (terrestrial). Bold p-values indicate p < 0.05.
(DOCX)

S8 Table Result of the comparison between groups for blunt flight behavior (predator and species) by Tukey’s test. Bold p-values indicate p < 0.05.
(DOCX)

S9 Table Summary of the model for retreat with cocking behavior. Intercept- Species (*B*. *jararaca*) and Predator (terrestrial). Bold p-values indicate p < 0.05.
(DOCX)

S10 Table Result of the comparison between groups for retreat with cocking behavior (predator and species) by Tukey’s test. Bold p-values indicate p < 0.05.
(DOCX)

S11 Table Summary of the model for strike-ready behavior. Intercept- Species (*B*. *jararaca*) and Predator (terrestrial). Bold p-values indicate p < 0.05.
(DOCX)

S12 Table Result of the comparison between groups for strike-ready behavior (predator and species) by Tukey’s test. Bold p-values indicate p < 0.05.
(DOCX)

S13 Table Summary of the model for flatten body behavior. Intercept- Species (*B*. *jararaca*) and Predator (terrestrial). Bold p-values indicate p < 0.05.
(DOCX)

S14 Table Result of the comparison between groups for flatten body behavior (predator and species) by Tukey’s test. Bold p-values indicate p < 0.05.
(DOCX)

S15 Table Summary of the model for gape behavior. Intercept- Species (*B*. *jararaca*) and Predator (terrestrial). Bold p-values indicate p < 0.05.
(DOCX)

S16 Table Result of the comparison between groups for gape behavior (predator and species) by Tukey’s test. Bold p-values indicate p < 0.05.
(DOCX)

S17 Table Summary of the model for strike behavior. Intercept- Species (*B*. *jararaca*) and Predator (terrestrial). Bold p-values indicate p < 0.05.
(DOCX)

S18 Table Result of the comparison between groups for strike behavior (predator and species) by Tukey’s test. Bold p-values indicate p < 0.05.
(DOCX)

S19 Table Data table. Frequencies of snake behaviors for each species and predatory model.
(XLSX)
