# Supplementary material for: Overcoming the phantoms of the past: Influence of predatory stimuli on the antipredator behavior of island pitvipers
Source: PLoS One. 2023 Oct 24;18(10):e0288826. doi: 10.1371/journal.pone.0288826 (PMC10597524; doi:10.1371/journal.pone.0288826)
Supplement: S1 Fig — (DOCX) [file pone.0288826.s001.docx]

**Supplementary material**


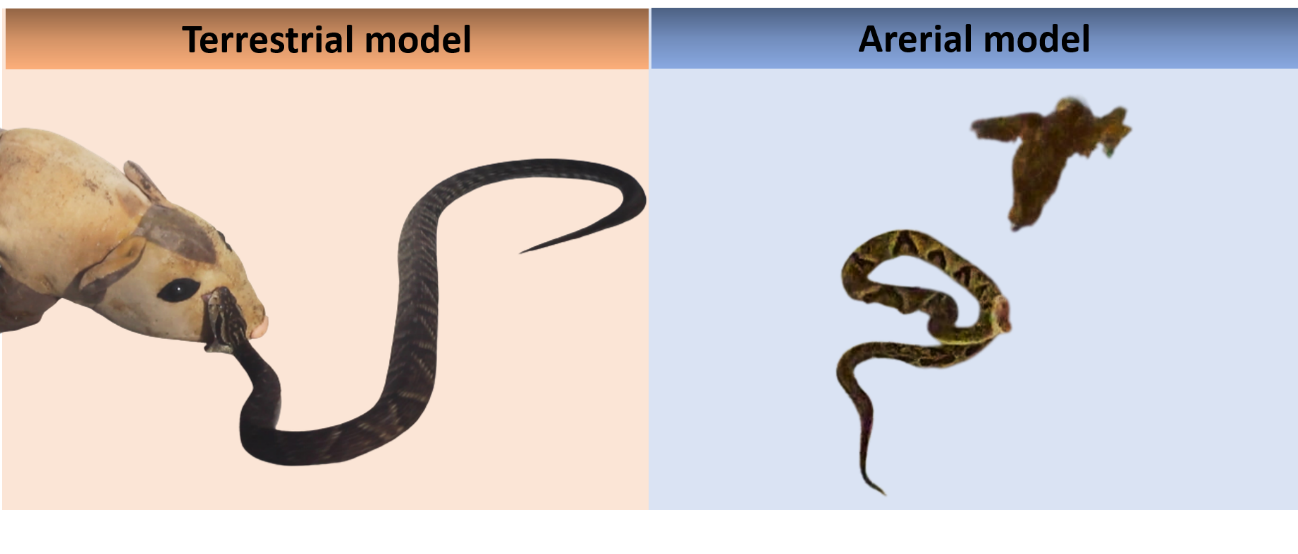


**S1 Figure.** Models of a terrestrial predator (Opossum: *Dideplphis albiventris*) and an aerial predator (taxidermied owl: *Athene cunicularia*)
