# Supplementary material for: Overcoming the phantoms of the past: Influence of predatory stimuli on the antipredator behavior of island pitvipers
Source: PLoS One. 2023 Oct 24;18(10):e0288826. doi: 10.1371/journal.pone.0288826 (PMC10597524; doi:10.1371/journal.pone.0288826)
Supplement: S11 Table — Intercept- Species (B. jararaca) and Predator (terrestrial). Bold p-values indicate p < 0.05. (DOCX) [file pone.0288826.s012.docx]

**Strike-ready**


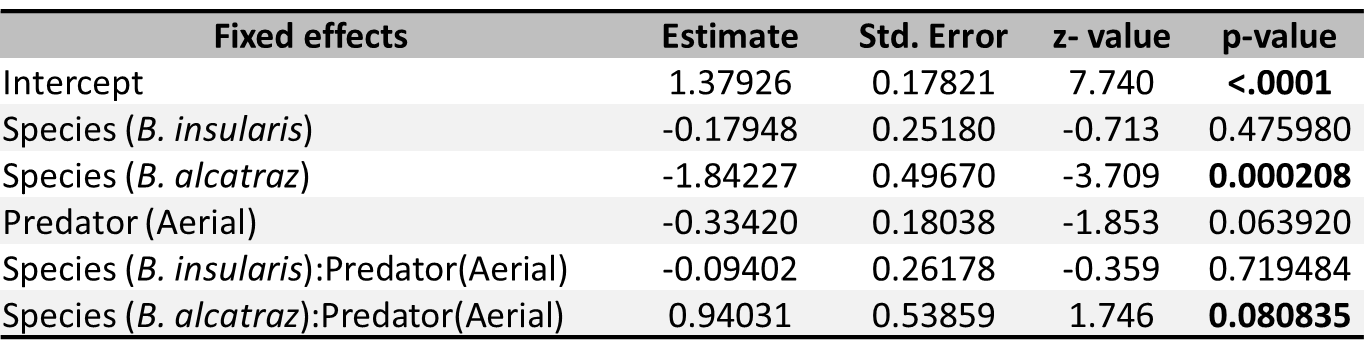


**S11 Table.** Summary of the model for strike-ready behavior. Intercept- Species (*B. jararaca*) and Predator (terrestrial). Bold p-values indicate p < 0.05.
