# Supplementary material for: Overcoming the phantoms of the past: Influence of predatory stimuli on the antipredator behavior of island pitvipers
Source: PLoS One. 2023 Oct 24;18(10):e0288826. doi: 10.1371/journal.pone.0288826 (PMC10597524; doi:10.1371/journal.pone.0288826)
Supplement: S14 Table — Bold p-values indicate p < 0.05. (DOCX) [file pone.0288826.s015.docx]

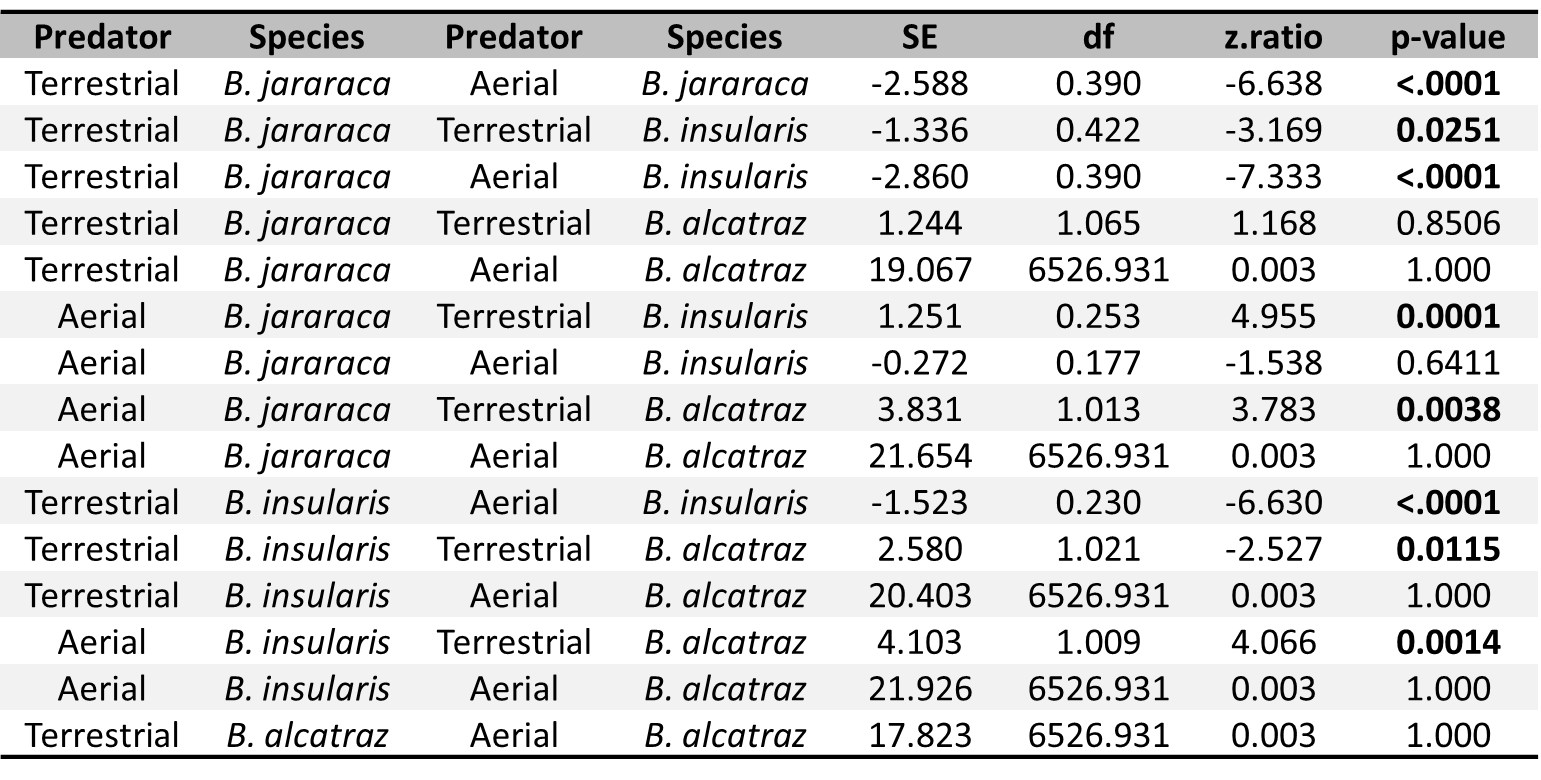


**S14 Table.** Result of the comparison between groups for flatten body behavior (predator and species) by Tukey's test. Bold p-values indicate p < 0.05.
